# Three-dimensional gait characteristics of patients after unilateral total knee arthroplasty

**DOI:** 10.1097/MD.0000000000026968

**Published:** 2021-08-27

**Authors:** Zong-Han Wang, Jin-Cheng Wang, Shuang Zheng, Pan Xue, Fu-Jun Han

**Affiliations:** a Department of the Oncology, Cancer Center, The First Hospital of Jilin University, Changchun, Jilin Province, China; b Department of the Orthopaedic, The Second Hospital of Jilin University, Changchun, Jilin Province, China.

**Keywords:** arthroplasty, gait analysis, knee, total knee arthroplasty

## Abstract

The purpose of this study is to evaluate the gait characteristics of bilateral limbs after unilateral total knee arthroplasty (TKA) using three-dimensional (3D) dynamic capture technology.

Forty-two patients who underwent TKA were selected from the Orthopedic Medical Center of The Second Hospital of Jilin University from November 2018 to May 2019. We used a 3D dynamic capture system to measure the gait characteristics of patients at 3 months after TKA. The data, including relative position and direction of different body parts, the force between feet and ground, spatial and temporal relationship of the lower limb muscles, were measured. Besides, the surface electromyogram signal and the force plate analog signal were also collected. The walking ability, knee 3D kinematic, and kinetic characteristics were analyzed by the Cortex software.

Spatial and temporal parameters, including stride frequency, double support phase, single support phase, step length, step time, step width, stride length, gait cycle, velocity, were no significant difference in bilateral lower extremities (*P* > .05). The reaction force of hip, knee, and ankle joint in the operation side were less than that of the healthy side, but the difference was not statistically significant (*P* > .05). However, when compared with the healthy side, the hip joint in operation side had a larger maximum extension angle (*P* < .001), the knee joint in operation side had a larger maximum valgus angle and valgus activity (*P* < .05), and had a smaller tibial maximum internal rotation angle (*P* < .05). Besides, the surface electromyogram signals of tibialis anterior muscles were reduced (*P* < .05).

3D gait analysis, as an objective and quantitative evaluation method, is a safe, effective, and reliable method for evaluating postoperative knee function. The data of gait analysis prove that TKA is a vital treatment to improve the function of patients with knee arthritis. Besides, gait analysis also showed that there were various kinematic and biomechanical abnormalities in the knee after TKA, which may be the reason why the surgical knee could not immediately return to normal level.

## Abstract 2

Supplemental Digital Content is available in the text

## 1 Introduction

Total knee arthroplasty (TKA) is an effective method for the treatment of advanced knee joint diseases, which can effectively correct knee deformity and improve knee function.^[1–5]^ However, TKA can lead to a partial inability of the knee joint to achieve a healthy knee joint movement. Therefore, accurate acquisition of gait information is an essential requirement for human kinematics research, and it is crucial to study the changes in joint kinematics and dynamics after artificial knee joint replacement.

Currently, the functional evaluation indicators for knee surgery include two-dimensional (2D) kinematic analysis, radiology, and functional scores. 2D motion analysis offers a more feasible and affordable alternative for dynamic knee valgus distance measurement.^[6,7]^ Nevertheless, 2D motion analysis has the disadvantage that many factors can affect distance measurement.^[7]^ X-ray, computer tomogram, and magnetic resonance imaging can be used to observe the angle of knee valgus and valgus, bone hyperplasia, and soft tissue swelling, respectively. However, radiographic imaging examination can only provide static images of the limbs, while being unable to reflect the most closely related motor function with the patient's symptoms and posttreatment satisfaction. In addition, commonly used functional scores include the American Knee Association Knee Score,^[8]^ the International Knee Documentation Committee Score,^[9]^ the Western Ontario and McMaster University Osteoarthritis Index,^[10]^ and so on. These function scores were based on questionnaires filled out by patients and then showed improvement in pain, knee range of motion, walking, and quality of life. However, these scoring methods have long been used in clinical practice and were susceptible to the training level of tester and patient compliance. Furthermore, the objectivity of the score results remains questionable.

Gait analysis, as a quantitative and objective evaluation tool for the lower extremities function, has made up for the shortcomings of 2D kinematic analysis, radiographic imaging examination, and functional scoring. It has been widely used in rehabilitation,^[11]^ neurology,^[12]^ children,^[13,14]^ and after knee surgery.^[3,15,16]^ We hypothesized that a successful TKA has many similarities of gait characteristics in the affected side and healthy side, including spatial and temporal parameters, similar reaction force of lower limb joints, and the maximum valgus angle and valgus activity of the knee joint. Therefore, the purpose of this study is to compare the gait characteristics of affected and healthy limbs after unilateral TKA using three-dimensional (3D) dynamic capture technology.

## 2 Methods

### 2.1 Ethics

The study was approved by the Institutional Review Board of the Second Hospital of Jilin University. The subjects were fully informed, signed consent, and voluntarily participated in the study.

### 2.2 Clinical background of the participants

Forty-two patients undergoing TKA were selected from the orthopedic medical center of The Second Hospital of Jilin University from November 2017 to May 2019. To improve the reliability of this study, we used the standard diagnostic criteria for unilateral knee arthritis. ① Repeated knee pain in the past month② Radiographs (standing or weight-bearing) show narrowing of joint space, subchondral sclerosis or cystic changes, and osteophyte formation at the joint margin; ③ Age ≥ 50 years; ④ Morning stiffness time ≤ 30 minutes; ⑤ Bone frictions when moving. Satisfying diagnostic criteria ① + (any 2 of ②, ③, ④, or ⑤) may lead to knee osteoarthritis diagnosis. Inclusion criteria of the experimental group: (1) Unilateral knee arthritis; (2) Eligible pre-operative symptoms and imaging; (3) Pre-operative hospital for special surgery score ranging from 12 to 74 and pre-operative Western Ontario and McMaster University Osteoarthritis Index (Table 1) ranging from 37 to 56; (4) No other lower limb diseases and surgical history; (5) No severe cardiopulmonary and neurological disorders; (6) Patients were fully informed and voluntarily participated in the study. Exclusion criteria of the experimental group: (1) Severe cardiopulmonary and cerebral diseases; (2) Psychological and mental abnormalities; (3) With other conditions restricting exercises; (4) Bilateral knee arthritis; (5) Participants with a previous knee injury. Two trained joint surgeons who did not understand the purpose of the study independently evaluated the patients.

**Table 1:** WOMAC index.

Pain	1. Walk on flat ground	0–1–2–3–4–5–6–7–8–9–10
	2. Up and down the stairs	
	3. When you go to bed at night	
	4. Sit up or lie down	
	5. Standing	
Stiffness	6. When you wake up in the morning	0–1–2–3–4–5–6–7–8–9–10
	7. Sitting, lying down, or resting for a period of time after waking up	
Physical function	8. When go downstairs	0–1–2–3–4–5–6–7–8–9–10
	9. When go upstairs	
	10. When you get up from your seat	
	11. When standing	
	12. When bending forward	
	13. When walking on flat ground	
	14. Getting in and out of a car or bus	
	15. When shopping	
	16. When wearing socks	
	17. Wake up	
	18. When taking off socks	
	19. Lying in the bed	
	20. When going in and out of the bathtub	
	21. When sitting	
	22. Sit on or stand up from the toilet	
	23. When doing heavy housework	
	24. When doing light housework	

The WOMAC scale evaluates knee function from 3 aspects: pain, stiffness, and physical function. There are 24 items, including 5 items for pain, 2 items for stiffness, and 17 items for physical function. Each item had a 10-point score, with patients marked according to their level of pain or functional limitations. In addition, higher WOMAC score indicates more severe osteoarthritis. Based on the total score, severity of osteoarthritis was assessed according to the following criteria: Mild < 80, Moderate 80–120, and Severe > 120. WOMAC = Western Ontario and McMaster University Osteoarthritis Index.

### 2.3 Device information

Motion Analysis (State Farm Dr Ste 100, CA) was used in this study, which consisted of 6 optical cameras (Beijing Feichuang Yida Photoelectric Technology Co., Ltd, China) located in different sites, high-precision force plates, and Cortex software. Regarding the Motion Analysis accuracy, the deviation of plane movement was less than 1 mm, and the deviation of rotational freedom was no more than 1.5°.

### 2.4 3D motion capture technology

The gait analysis was performed by a skilled physician. The doors and windows were closed, and the curtains were pulled off to prevent outside light. All the glowing objects in the room were cleared to avoid the interference of external light points. The temperature in the room was adjusted to 25 to 28 °C. The patients took off their shoes and socks and put on the tights for the experiment. The height, body mass, length of the lower legs (the distance from the anterior superior iliac spine to the medial malleolus), the knee width (the width between the inner and outer edge of the knee), and the ankle width (the width between the inner and outer jaws) were measured.

**Figure 1:**
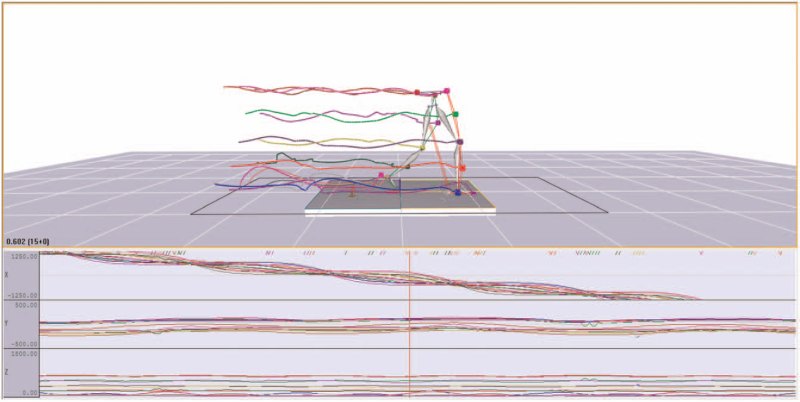
Construction of virtual 3D lower limb Helen-Haynes model. This picture shows the movement of lower limbs. The marker balls were used to mark the lower limbs. The figure showed 15 points to construct the virtual lower extremites model. 3D = three-dimensional.

Reflective marker balls of 14 mm in diameter were used for body marking. A total of 19 and 15 marking sites for the dynamic and static date collection were selected according to the system module. Then, the marker balls were attached to the sites, including the left and right sides of the anterior superior iliac spine, the posterior superior iliac spine, the lower 1/3 of the lateral thigh (slightly lower than where the hand swung by), upper knee lateral epicondyle, lower 1/3 of lateral shank, apex of lateral malleolus, second metatarsal head and heel (at the same height as the second metatarsal head) (Fig. 1).

**Figure 2:**
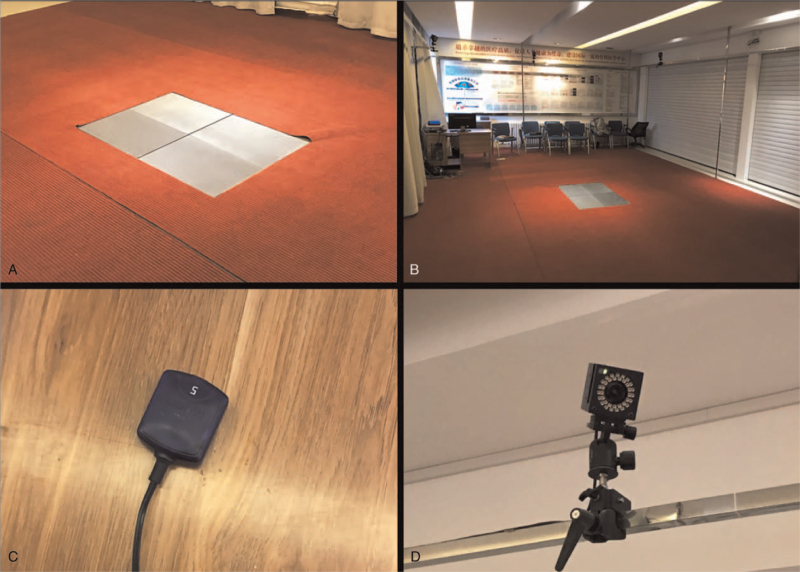
Spatial layout of 3D dynamic capture device. (A) Four three-dimensional force plates were embedded in the vertical direction in the ground, (B) the gait analysis room, (C) the surface EMG device and (D) optical cameras. 3D = three-dimensional, EMG = electromyogram.

**Figure 3:**
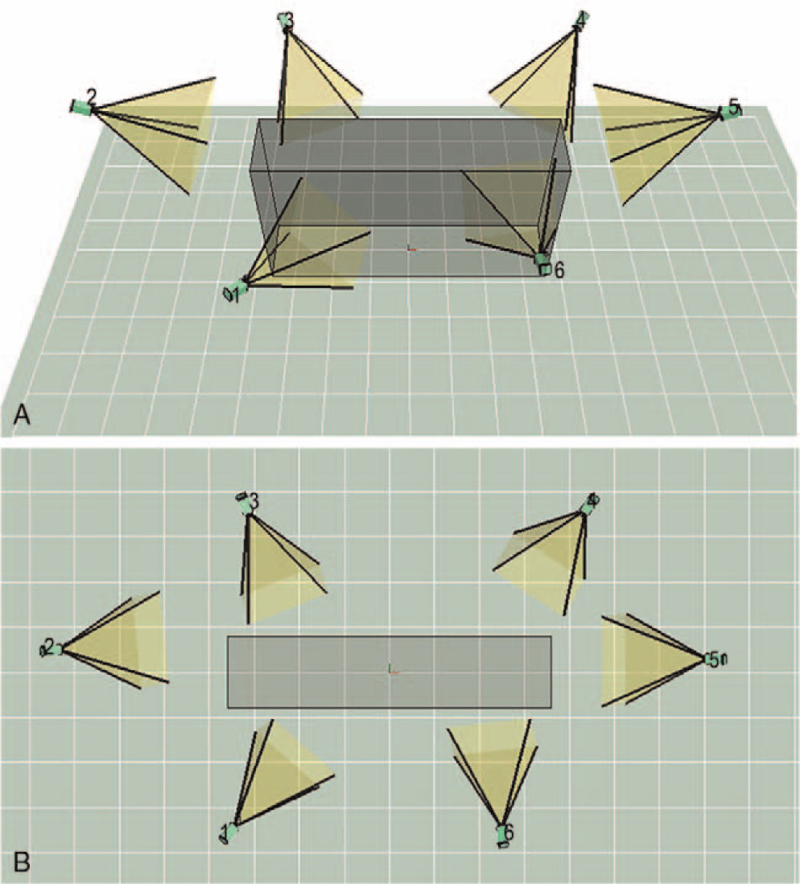
Position relationship between optical cameras. (A) The 3D view, (B) the vertical view. 3D = three-dimensional.

**Figure 4:**
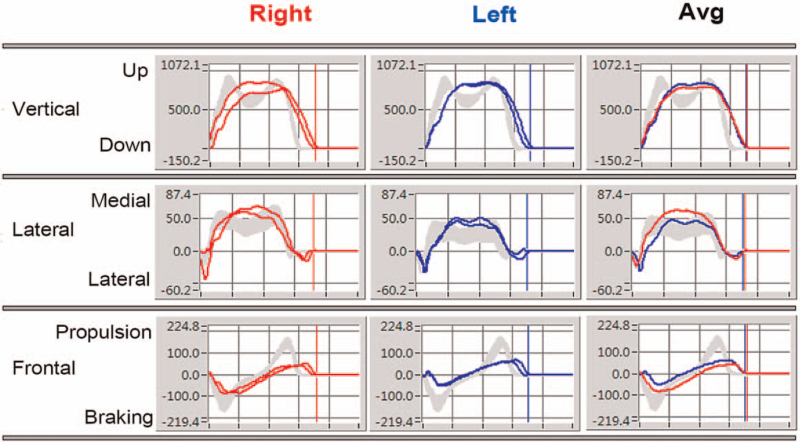
A 62-year-old female patient was diagnosed with right knee osteoarthritis and underwent right TKA. The results of gait analysis at 3 months after operation showed that there was no significant difference in the reaction between the feet and the ground. TKA = total knee arthroplasty.

The host and infrared cameras were configured, with 6 infrared cameras (frequency set to 100 Hz) fixed on the laboratory wall, 4 3D force plates embedded in the vertical direction in the ground (Figs. 2–3). The patients keep head up, look straight ahead, and have the feet as wide as shoulders. When the patients kept the standard standing position, the spatial origins of the mark points were recorded by the system, and a static model was established. Then, the patient was told to walk straight on the plates in their usual manner and speed, to ensure that each foot was stepped on a force plate during walking, and to walk for at least 10 times (Fig. 4). The walks of high-quality images and natural gait were selected from the dynamic acquisition. Three times of walk were chosen for each patient, and 1 gait cycle on the force plate of each foot was intercepted from each step. The average data of the lower limbs of the patients were compared between operation side and contralateral side.

The spatial and temporal parameters, knee angle, angular velocity, knee moment, ground reaction force (internal and external, front and back, vertical) were recorded. Besides, we also collected surface electromyogram (EMG) signals from the gluteus maximus, vastus medialis, hamstring, gastrocnemius, and tibialis anterior muscles.

### 2.5 Statistical process

Statistical analyses were performed using SPSS software version 20.0 (IBM SPSS, IBM Corp., Armonk, NY). The method was the *t* test and correlation analysis of independent samples. Significance was defined as *P* < .05. Data values were presented as mean ± SD.

## 3 Results

### 3.1 Clinical background of the participants

The average age was 60.3 years old (range from 50–79 years old). The subjects included 7 male patients with an average height of 172 ± 12 cm and an average weight of 60 ± 13 kg, and 35 female patients, with an average height of 163 ± 9 cm and an average weight of 51 ± 11 kg. The kinematic and kinetic parameters were measured at 3 months after TKA. The screening of participants and radiological assessment was done by 2 trained joint surgeons who did not understand the purpose of the study. The 2 trained surgeons independently performed the evaluation. If the evaluated results are consistent, the patient was enrolled. However, if there were any disagreements, all of the authors discussed until consensus could be reached.

### 3.2 Spatial and temporal parameters

The stride frequency, double support phase, single support phase, step length, step time, step width, stride length, gait cycle, and velocity, were no significant difference between affected and healthy limbs (*P* > .05) (Table 2).

**Table 2:** Results of spatial and temporal parameters of gait analysis after operation of bilateral lower limbs.

	OS (mean ± SD)	HS (mean ± SD)	*P*
Stride length (cm)	49.10 ± 3.13	46.38 ± 2.88	.376
Step length (cm)	97.19 ± 6.54	98.32 ± 5.69	.789
Average forward speed (cm/s)	80.03 ± 9.61	80.57 ± 9.40	.930
Average step frequency (step/min)	99.22 ± 10.00	98.10 ± 9.21	.851
Total support duration (%)	61.32 ± 1.74	62.52 ± 2.62	.372
Pendulum dynamic duration (%)	11.19 ± 2.66	11.96 ± 2.05	.372
Duration of initial bipedal support (%)	35.46 ± 2.62	11.96 ± 2.03	.602
Single leg support time (%)	35.46 ± 2.62	36.66 ± 1.74	.372

HS = healthy side, OS = operation side.

### 3.3 Peak value of joint reaction force

The reaction force of hip, knee, and ankle joint in the operation side were less than that of the healthy side, but the difference was not statistically significant (*P* > .05) (Table 3).

**Table 3:** Results of peak joint reaction force in gait analysis.

	OS (mean ± SD)	HS (mean ± SD)	*P*
The reaction force of hip joint (N/kg)	50.8 ± 27.7	49.4 ± 15.2	.892
The reaction force of knee joint (N/kg)	42.4 ± 16.4	45.2 ± 20.1	.738
The reaction force of ankle joint (N/kg)	66.8 ± 25.8	89.1 ± 24.8	.061

HS = healthy side, OS = operation side.

### 3.4 The ROM of hip, knee, and ankle joint

Regarding the hip joint, the maximum flexion angle, maximum extention angle, maximum internal rotation angle, and range of motion (ROM) of the operative side were smaller than those of the contralateral side (*P* < .05). However, there was no statistical difference in the range of flexion and extension, and the maximum external rotation angle (Table 4).

**Table 4:** The ROM of the hip joint.

	OS (mean ± SD)	HS (mean ± SD)	*P*
Flexion and extension			
Maximum flexion angle (°)	–2.6 ± 5.6	2.5 ± 5.5	.038
Maximum extension angle (°)	–38.0 ± 2.7	–33.2 ± 3.1	<.001
Range of motion (°)	35.2 ± 3.1	36.0 ± 4.2	.676
Internal and external rotation			
Maximum internal rotation angle (°)	5.4 ± 14.3	36.59 ± 4.2	.435
Maximum external rotation angle (°)	–8.6 ± 14.8	2.5 ± 5.5	<.001
Range of motion (°)	14.8 ± 5.6	36.54 ± 4.2	<.001

In flexion and extension, negative value represents extension and positive value indicates flexion. In internal and external rotation, negative value represents internal rotation and positive value indicates external rotation. HS = healthy side, OS = operation side, ROM = range of motion.

Concerning the knee joint, the maximum extention angle, maximum valgus angle, and ROM of varus and valgus in the operation side were larger than those of the contralateral side (*P* < .05). Besides, the maximum internal torsion angle of tibia was significantly reduced on the operative side (*P* < .05). Nevertheless, there was no difference in the maximum flexion angle, flexion and extension activity, maximum external torsion angle (*P* > .05) (Table 5).

**Table 5:** The ROM of the knee joint.

	OS (mean ± SD)	HS (mean ± SD)	*P*
Flexion and extension			
Maximum flexion angle (°)	66.7 ± 2.9	64.7 ± 3.9	.185
Maximum extension angle (°)	14.5 ± 3.0	10.9 ± 1.7	<.001
Range of motion (°)	51.1 ± 3.3	5.6 ± 5.1	.193
Internal and external torsion			
Maximum internal torsion angle (°)	10.5 ± 11.6	23.2 ± 1.8	.024
Maximum external torsion angle (°)	–25.5 ± 16.1	–16.7 ± 4.5	.238
Range of motion (°)	33.7 ± 17.1	42.1 ± 4.5	.224
Varus and valgus			
Maximum varus angle (°)	–0.5 ± 2.7	–1.5 ± 3.4	.582
Maximum valgus angle (°)	15.2 ± 7.2	6.9 ± 4.6	.021
Range of motion (°)	15.8 ± 4.4	8.6 ± 2.3	.002

Internal and external torsion indicates the tibia rotation with reference to the femur, the negative value represents external torsion and a positive value suggests internal torsion. In varus and valgus, the negative value represents varus and a positive value indicates valgus. HS = healthy side, OS = operation side, ROM = range of motion.

As for the ankle joint and the subtalar joint, the maximum valgus angle and the maximum varus angle of the subtalar joint in the operative side were larger than that of the contralateral side (*P* < .05). In addition, the ROM of the operative side was decreased (*P* < .05) (Table 6).

**Table 6:** The ROM of ankle and subtalar.

	OS (mean ± SD)	HS (mean ± SD)	*P*
Ankle flexion and extension			
Maximum dorsiflexion angle (°)	32.3 ± 5.1	31.1 ± 4.8	.285
Maximum plantar flexion angle (°)	2.7 ± 5.2	2.1 ± 5.3	.790
Range of motion (°)	30.7 ± 3.1	29.7 ± 2.1	.150
Subtalar varus and valgus			
Maximum valgus angle (°)	3.6 ± 10.1	–6.3 ± 6.5	.011
Maximum varus angle (°)	–19.4 ± 9.8	–54.1 ± 10.9	<.001
Range of motion (°)	23.1 ± 3.1	47.7 ± 10.5	<.001

In ankle flexion and extension, negative value indicates dorsiflexion and positive value indicates plantar flexion. In subtalar varus and valgus, negative value represents varus and positive value indicates valgus. HS = healthy side, OS = operation side, ROM = range of motion.

### 3.5 Surface EMG signals

The surface EMG signals of tibialis anterior muscle (*P* < .05) and gastrocnemius muscle (*P* > .05) in the operation side were decreased. Other EMG recordings, including gluteus maximus, vastus medialis, hamstring, and gastrocnemius, revealed no significant difference (*P* > .05) (Table 7).

**Table 7:** Peak value of surface EMG signals.

	OS (mean ± SD)	HS (mean ± SD)	*P*
Gluteus maximus	2.31 ± 1.72	2.30 ± 1.58	.978
Vastus medialis	1.52 ± 1.20	1.15 ± 0.61	.371
Hamstring	2.45 ± 1.76	1.79 ± 0.88	.676
Tibialis anterior muscle	2.27 ± 0.70	3.29 ± 1.34	.035
Gastrocnemius muscle	1.75 ± 0.72	2.32 ± 1.73	.328

EMG = electromyogram, HS = healthy side, OS = operation side.

## 4 Discussion

In this study, the results of 3D gait analysis were used to compare the differences in gait parameters between the healthy limb and the affected limb after TKA. Overall, 3D gait analysis is an objective, quantitative, and scientific tool for assessing knee function after TKA.

The commonly used gait information acquisition method is a 3D gait analysis system based on infrared tracking technology, which is usually composed of 3 parts: a motion capture system for capturing a 3D motion trajectory of a reflective marker, force plate for collecting ground reacting forces, and surface EMG.^[15]^ The camera captures the 3D coordinates of the human body's various landmarks in space and correlates with the digits in the digitized human biomechanical model to calculate the joint activity trajectory; the force gauge is generally level with the ground when the subject walks over the force plate can record the force and direction of the sole to the ground; the surface EMG system is attached to the skin surface through the electrode sheet, and records the main muscle EMG signals at different time points during walking. The data that are collected includes the relative position and direction of various parts of the body, the force of the foot and the ground, the temporal and spatial relationship, and the phased activities of the lower limb muscles.^[15,17–19]^ The 3 systems are integrated through computer software to maintain consistency in data collection for exercise, force, and muscle activity during the gait cycle.

The most important advantage of this technology is its non-invasive, easy to operate, and high patient fit properties. Secondly, not only the kinematics of a single joint can be acquired through 3D motion capture, but also the movement of multiple joints and the entire torso according to the layout of the body surface points. However, the most significant disadvantage of this technique is that the marking points are attached to the surface of the skin. During the movement, the sliding of the soft tissue will bring a tremendous error to the subsequent operation, and the joint error with greater flexion and extension activity is larger, and excellent measurement cannot be achieved. Consequently, only approximate kinematic data can be obtained.

The knee joint is a relatively complex joint of the human body.^[2,3,5,8–10,15,17,19]^ It needs to complete a wide range of joint movements under the condition of carrying human body gravity to meet the needs of human body movement. Therefore, the knee joint is the joint most commonly involved in osteoarthritis.^[20]^ TKA is a standard treatment for end-stage knee disease and works well.^[2–5,8,15]^ The walking function is the most essential part of daily life activities. TKA can significantly improve the limb function of patients, relieve pain, and improve the quality of life.^[2–5,8]^ Although the gait will be substantially improved after TKA, there are still some abnormalities that cannot be repaired for a long time after surgery. This is related to pre-operative joint stiffness, muscle strength, prosthetic geometry, surgical technique, and rehabilitation strategies. Gait analysis can help us better understand the mechanism of limb movement and improve the surgical outcome by developing correct muscle training strategies such as muscle strength.^[21]^ Chang et al^[22]^ found that the total number of steps per day of walking after TKA was positively correlated with patient function, so proper gait were essential for long-term tasks.

We found a significant difference between the affected limb and healthy limb, and we believe that gait examination is simple and easy and is an essential objective evaluation index before and after TKA. For a significant gait analysis comparison, we specially selected patients with unilateral knee osteoarthritis. Moreover, to rule out the influence factors, we excluded the patients with rheumatoid arthritis, severe internal and external valgus deformities, the history of TKA surgery, or other bone and joint disease. The results of the evaluation showed that the TKA procedure did not significantly change the spatial-temporal parameters of the gait. There was no statistical difference between the affected side and the good side of all the indicators, suggesting that the essential walking function of the patient has been relatively excellent postoperatively. Functional recovery is close to the average level of the healthy knee. However, TKA has a significant effect on the movement of the hip, knee and ankle joints. In this study, we believe that changes in knee flexion, extension mobility, and internal and external valgus activity are the most direct and most significant manifestations of TKA surgery on limb function. The maximal extension of the knee joint is significantly reduced. There is a certain degree of straightening dysfunction relative to the healthy side. Although the gait was evaluated at 12 months postoperatively, the knee extension function has not recovered to the normal state of the natural knee joint. The knee valgus angle of the affected side was significantly larger than that of the healthy side, suggesting that there is obvious instability of the internal and external valgus in the knee joint after TKA, which may be related to the damage of soft tissue in joint replacement surgery.

The range of rotation of the ipsilateral humerus is reduced, but the measurement results of the index on the operated side and the affected side are larger than the amplitude of the knee joint rotation recorded in the literature. We believe that it may be an error caused by the sliding of the skin surface marker point, but the result can be to some extent, it reflects the decrease in joint flexibility after TKA. The changes in the gait parameters of the hip and ankle joints are secondary changes in the flexion and extension of the knee joint. The knee joint is reduced in straightness, and the hip flexion amplitude is correspondingly reduced during the forward step, while the extension angle is increased. The above results suggest that TKA can affect the kinematics of the knee not only joint, but also further affects the kinematics of other joints of the lower extremities.

The surface EMG signals showed no difference in the bilateral gluteus maximal signals. The medial and hamstring muscles were enhanced on the affected side, while the tibialis anterior and gastrocnemius muscles were weakened on the surgical side. The signal enhancement of the medial and hamstring muscles suggests that the patient needs to mobilize more muscle fibers to participate in exercise during walking, which is a manifestation of the decrease in muscle work efficiency. At the same time, the hamstring muscle is an antagonist of the quadriceps muscle. The reduction in joint dynamic stability,^[23]^ this muscle potency reduction, is generally attributed to the destruction of the joint structure by surgical trauma. The tendency of the tibialis anterior and gastrocnemius muscle activities to decrease on the affected side is similar to that reported in other literature.^[16]^

In theory, different subjects should walk at the same speed to reduce the influence caused by the difference in walking speed. Therefore, to obtain the kinematic parameters of the natural state, we set the pace in advance. According to the previous studies, the reduction of knee mobility after TKA is the most common change, and patients have reduced joint mobility in both the swing phase and the support phase.^[3,24]^ Joint mobility is one of the most important indicators for determining postoperative function.^[25]^ However, in clinical examinations, the passive activity of patients usually does not decrease, and the reduction of active activity is not as significant as the results of gait analysis. The relationship between activity reduction and limb function in gait analysis is still not well interpreted, and follow-up studies should focus on the interpretation of gait analysis results and clinical relevance. In addition, systematic review^[26]^ has found that the effects of different studies are seriously inconsistent, which may be related to the differences in data collection methods and prosthesis design. In our opinion, to improve the clinical application value of gait analysis, long-term research should strive to achieve methodological unification.

### 4.1 Limitations of this study

Although gait analysis provides a reliable basis for the evaluation of postoperative knee joint function, there are remain several limitations. First, gait analysis results might vary between different ages and genders. There are also significant differences between different heights and races. Secondly, the results of gait analysis can also be influenced by the hardware equipment, software analysis process, and the technical level of testers. To date, it is difficult to form a unified standard, especially the interpretation of the results. Thirdly, this study enrolled relatively small participants and paucity healthy population as a control group. Finally, this study only carried out gait tests for a sport that was walking on the ground. Other sports, such as up and downstairs, squatting, and turning back also had an impact on daily activities, were not tested. Thus, it is necessary to establish an extensive sample gait database before large-scale gait examination and determine the standard reference value of each parameter according to age group and gender. Moreover, subsequent studies will include more cases and gradually explore the best experimental protocols to maximize the benefits of gait analysis results.

## 5 Conclusion

3D gait analysis, as an objective and quantitative evaluation method, is a safe, effective, and quantitative method for evaluating postoperative knee function. The data of gait analysis prove that TKA is a vital treatment to improve the function of patients with knee arthritis. Besides, gait analysis also showed that there were various kinematic and biomechanical abnormalities in the knee after TKA, which may be the reason why the surgical knee could not immediately return to normal level.

Supplemental Digital Content, http://links.lww.com/MD/G370.

## Author contributions

**Conceptualization:** Jin-Cheng Wang.

**Methodology:** Zong-Han Wang.

**Supervision:** Fu-Jun Han, Jin-Cheng Wang.

**Validation:** Pan Xue, Jin-Cheng Wang.

**Visualization:** Fu-Jun Han.

**Writing – original draft:** Zong-Han Wang, Shuang Zheng.

**Writing – review & editing:** Jin-Cheng Wang.
